# Highly Efficient Tribocatalysis of Superhard SiC for Water Purification

**DOI:** 10.3390/nano15151206

**Published:** 2025-08-06

**Authors:** Yuanfang Wang, Zheng Wu, Siqi Hong, Ziqi Zhu, Siqi Wu, Biao Chen, Yanmin Jia

**Affiliations:** 1 Xi’an Key Laboratory of Textile Chemical Engineering Auxiliaries, School of Environmental and Chemical Engineering, Xi’an Polytechnic University, Xi’an 710048, China; 15103591188@163.com (Y.W.); hongsiqi@xpu.edu.cn (S.H.); 13753786523@163.com (Z.Z.); wusiqiyjs@163.com (S.W.); 2 Quantum Materials and Devices Key Laboratory of Shaanxi Province’s High Education Institution, School of Physics and Information Technology, Shaanxi Normal University, Xi’an 710119, China; chenbiao@snnu.edu.cn

**Keywords:** tribocatalysis, dye decomposition, silicon carbide, harvesting friction energy

## Abstract

Mechanical friction offers a frequent approach for sustainable energy harvesting, as it can be captured and transformed into electricity by means of the triboelectric phenomenon. Theoretically, this electricity may subsequently be employed to drive electrochemical water purification processes. Herein, the experimental results confirm that the SiC particles effectively trigger the tribocatalytic decomposition of Rhodamine B (RhB). During the tribocatalytic decomposition of dye, mechanical friction is generated at the contact surface between the tribocatalyst and a custom-fabricated polytetrafluoroethylene (PTFE) rotating disk, under varying conditions of stirring speed, temperature, and pH value. Hydroxyl radicals and superoxide radicals are confirmed as the dominant reactive species participating in tribocatalytic dye decomposition, as demonstrated by reactive species inhibition experiments. Furthermore, the SiC particles demonstrate remarkable reusability, even after being subjected to five consecutive recycling processes. The exceptional tribocatalytic performance of SiC particles makes them potentially applicable in water purification by harnessing environmental friction energy.

## 1. Introduction

The quality of water consumed is essential for maintaining fundamental physiological processes, such as the distribution of nutrients and the regulation of temperature, acid–base balance, and electrolytes within the body. Nevertheless, the rapid industrial expansion in recent decades has significantly contributed to the persistent issue of water pollution, particularly due to the organic dye effluents generated by the textile sector, which contribute significantly to environmental contamination and represent a serious risk to human health [1,2,3,4,5]. Organic dyes are extensively employed in the manufacturing of textiles, printing materials, and plastic products. However, these synthetic colorants exhibit significant toxicity and are linked to carcinogenic and mutagenic effects, posing serious risks to various forms of life [6]. Contaminated water has a high concentration of organic matter and a complex composition and is difficult to decompose naturally [7,8,9,10]. Water purification has historically relied on chemical oxidation, microbial degradation, and physical separation techniques [11,12,13]. For example, traditional physical adsorption techniques primarily transfer organic contaminants from aqueous solutions to adsorbent materials without enabling the direct decomposition of dye-laden wastewater [12,14]. The biological approach can effectively purify water using microorganisms; however, its real-world application is constrained by strict operational conditions, including sensitivity to pH, temperature, and the specific nature of pollutants in the water [15,16]. Physical purification approaches rely on adsorption to relocate toxic compounds from water to another phase, without degrading them, thereby posing a risk of secondary pollution [17].

Tribocatalysis offers a compelling route for transforming mechanical input into chemical output and has been recognized as a reliable technique for utilizing mechanical forces in energy conversion [18,19,20,21,22]. This technique shows promise for diverse applications, including the production of combustible gases [21], water splitting for hydrogen production [23], and the rehabilitation of degraded water systems [24,25]. Based on the mechanism of triboelectrification, mechanical interaction between two dissimilar materials induces surface charge separation, with opposite charges accumulating on each material upon contact [21,26]. In tribocatalysis, a material may function as a catalyst even without possessing semiconducting properties. Furthermore, the polarity of the charge generated on a frictional surface can be anticipated based on its position in the triboelectric series [26]. Consequently, once triboelectrically generated surface charges are introduced into the redox system, they may interact with dissolved oxygen and hydroxyl groups to generate reactive oxygen species, which subsequently initiate a series of redox reactions [20, 21, 22]. Since tribocatalysis entails only physical and chemical interactions at the catalyst’s surface, its intrinsic electrical properties are not essential, thereby enabling a wide variety of materials to serve as potential tribocatalysts. In 2019, Li et al. already demonstrated the use of Ba_0.75_Sr_0.25_TiO_3_ nanoparticles [23] to achieve dye decomposition through the utilization of ambient mechanical energy. A range of catalysts have since been developed, including Ba_4_Nd_2_Fe_2_Nb_8_O_30_ [19], CdS [20], TiO_2_ [24], ZnO [25], BiOIO_3_ [27], Bi_12_TiO_20_ [28], Bi_2_WO_6_ [29], Ba_2_._5_Sr_2_._5_Nb_8_Ta_2_O_30_ [30], SrTiO_3_ [31] and so on, and have been explored for environmental remediation through tribocatalytic processes. However, most tribocatalytic materials developed so far encounter notable limitations, such as low catalytic performance, elevated fabrication expenses, and the risk of producing secondary contaminants due to the incorporation of environmentally unfriendly particulate substances. Therefore, tribocatalysts that exhibit high efficiency, economic feasibility, and environmental sustainability are highly suitable for practical implementation. Nevertheless, the design and development of innovative tribocatalytic materials remain significant challenges in advancing the field.

Silicon carbide (SiC), recognized as a representative third-generation semiconductor due to its abundant availability, robust physicochemical stability, and excellent resistance to both acidic and alkaline environments [32,33], has attracted growing interest across diverse applications, such as catalysis [34,35], photodetection [36], supercapacitive energy storage [32], and electromagnetic wave attenuation [37]. The hardness of SiC is extremely high, with a Moh’s hardness of 9.2 to 9.5, which is the fourth highest hardness of any known material, second only to that of diamond [38]. During friction, SiC particles generate scratches on the polytetrafluoroethylene (PTFE) surface because SiC particles are much harder than PTFE [39]. The PTFE has more active surfaces exposed by the scratches [40]. Specifically, the introduction of scratches increases the contact surface of PTFE, exposing a greater portion of the active area of the PTFE [41]. This enhancement leads to greater electrostatic accumulation across the PTFE contact boundary [42].

The scratches generated during the friction catalysis process are of significance: on the one hand, they significantly enlarge the effective contact area, thereby enhancing the interfacial interactions between PTFE and the reactants, and on the other hand, they expose high-energy surfaces within the bulk material, which is conducive to the occurrence of chemical reactions. More importantly, due to the increased contact area and the activation of interfacial structure, the PTFE surface is able to accumulate more triboelectric charges during the sliding process, thereby strengthening the local electric field intensity at the interface [43]. This accumulation of surface charges not only regulates the interfacial energy states but also drives the separation and migration of electrons [44].

Within redox systems, friction-induced interfacial charge buildup and localized electric fields can greatly enhance electron transfer dynamics, thereby reducing the activation energy required for interfacial reactions [45]. Meanwhile, the scratching effect induced by the SiC particles also enhance the adsorption and activation abilities of the reactants. The synergistic effect of scratching, triboelectric effect, and surface activity effectively improves the overall catalytic efficiency of the redox reaction, where electron transfer is the rate-controlling step. Therefore, the friction system constructed by SiC and PTFE provides a new strategy and pathway for friction-induced redox catalysis by realizing a “mechanical–charge–interface” triple coupling mechanism.

In this work, Rhodamine B (RhB) dye was successfully decomposed by friction between the PTFE rotating disk and the surface of SiC particles through mechanical stirring. The high hardness SiC particles can effectively cut and disrupt the PTFE surface during the friction process, which promotes electron transfer and friction charge generation. This efficient charge generation mechanism can significantly increase the tribocatalytic dye decomposition ratio. After 4 h of stirring, the SiC particles can decompose 98.9% of the RhB dye in the tribocatalysis process. This study systematically investigates the influence of critical variables, such as stirring speed, reaction temperature, and pH value, to identify the most favorable parameters governing tribocatalytic activity. The SiC particles exhibiting outstanding tribocatalytic activity demonstrate strong potential for future use in water purification by harnessing ambient mechanical energy.

## 2. Materials and Methods

### 2.1. Materials

The SiC particles (1 μm) used in this experiment were a commercially available material purchased from Shanghai Yige Alloy Co., Ltd. (Shanghai, China). All experiments were performed using deionized water, with the principal compounds being rhodamine B (RhB, C_28_H_31_C_l_N_2_O_3_), methylene blue (MB, C_16_H_18_C_l_N_3_S), methyl orange (MO, C_14_H_14_N_3_SO_3_Na), hydrochloric acid (HCl), sodium hydroxide (NaOH), methanol (CH_4_O), ethylene diamine tetra-acetic acid (EDTA, C_10_H_16_N_2_O_8_), tert-butyl alcohol (TBA, C_4_H_10_O), benzoquinone (BQ, C_6_H_4_O_2_), and silver nitrate (AgNO_3_); the aforementioned compounds were acquired from Sinopharm Chemical Reagent Co., Ltd., Shanghai, China. The chemicals used were of analytical-grade (AR) purity, suitable for analytical applications.

### 2.2. SiC Particle Synthesis

Ball milling represents a conventional top-down strategy widely employed for the mass production of functional materials [46,47]. High-performance micro SiC particles were prepared by the method of ball milling, taking low grade coarse SiC particles as raw materials. The SiC particles were mechanically alloyed in a PULVERIZER 80 planetary mill using a zirconium oxide vessel. The milling balls, with a diameter of 5 mm, were also composed of zirconium oxide. To prevent the agglomeration and caking of SiC particles during dry ball milling, a wet milling method was employed, with ethanol used as the process control agent (PCA). The initial SiC particles were ball-milled for several hours.

### 2.3. Material Characterization

X-ray diffraction (XRD, model DX-2700, Haoyuan Instrument, Dandong, China) was executed to characterize the crystal structure of the synthesized materials. Scanning electron microscopy (SEM, Nova NanoSEM X30, Thermo Fisher Scientific, Winsford, UK) was utilized to the analysis of surface topography and elemental composition of the materials. A UV–Vis spectrophotometer (UV2300II, Techcomp, Shanghai, China) was utilized to record the absorbance spectra of the RhB solution. To investigate the active radical species produced through tribocatalysis, an electron paramagnetic resonance spectrometer (EPR, CIQTEK EPR200M, CIQTEK, Hefei, China) was utilized.

### 2.4. Tribocatalytic Decomposition of RhB Using SiC Particles

In a standard experimental setup, 200 mg of the SiC particles were dispensed into a φ 45 × 60 mm glass beaker with a non-smooth inner surface, followed by the addition of 30 mL RhB aqueous solution (10 mg/L). Mechanical agitation was performed using a custom-fabricated PTFE rotating disk operating at 600 rpm at ambient temperature under dark conditions. For the purpose of monitoring the dye decomposition behavior, 2 mL aliquots were extracted at designated intervals and centrifuged to separate the supernatant. A UV–Vis spectrophotometer was employed to analyze the absorbance spectra of the RhB solution.

### 2.5. Activity Detection

To elucidate the mechanism governing the tribocatalytic decomposition of dye under varying conditions, a series of scavenger-based experiments were implemented as previously reported [11,44]. Each scavenger (1 mmol EDTA, BQ, TBA, or AgNO_3_) was separately added to a reaction system composed of 200 mg SiC particles and 30 mL of RhB solution (10 mg/L). These agents were employed to selectively inhibit holes (h^+^), superoxide radicals (·O_2_^−^), hydroxyl radicals (·OH), and electrons (e^−^), respectively [48,49]. The tribocatalytic performance was then assessed under identical conditions to those used in the baseline dye decomposition experiments.

An EPR analysis was performed using a spin-trapping strategy, in which an unsaturated diamagnetic compound was introduced into the reaction system to capture short-lived radical intermediates. The resulting radical–adduct complexes produce characteristic signals detectable by the EPR spectrometer. To detect ·OH and ·O_2_^−^ species, 3 mL of deionized water and methanol were, respectively, added to a mixture of 50 μL DMPO and 200 mg of SiC particles, which was then subjected to tribocatalytic treatment for 1 h. The final solution was analyzed to identify the reactive species generated during the reaction.

## 3. Results

**Figure 1:**
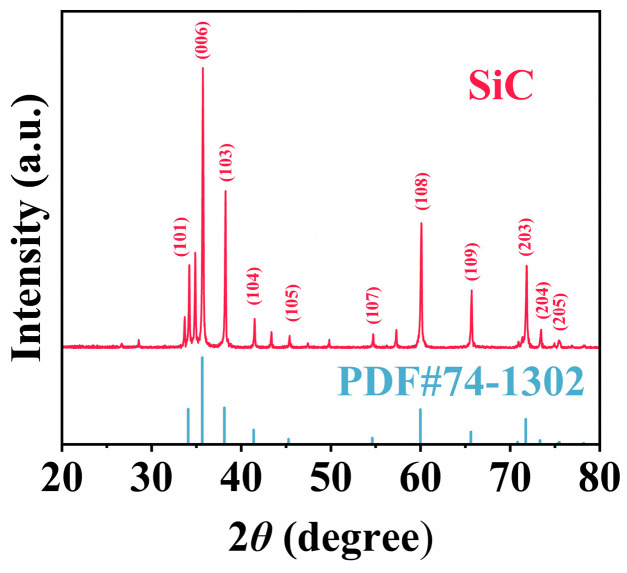
XRD pattern of the SiC particles.

According to Figure 1, based on the XRD observations, the characteristic peaks are located at 34.1°, 35.6°, 38.1°, 41.4°, 45.3°, 54.6°, 60.0°, 65.6°, 73.3°, 75.5° and 78.2°, corresponding to the (101), (006), (103), (104), (105), (107), (108), (109), (203), (204) and (205) crystal planes, respectively. The measured diffraction reflections correspond well to the standard SiC pattern (PDF#74-1302), indicating that the SiC particles possess a distinct hexagonal crystal phase with the *P6_3_mc* space group [50].

From the XRD pattern, a slight shift in the (101) diffraction peak can be observed, which may be attributed to internal stress, lattice distortion, or microstrain induced during the synthesis process. The appearance of an additional weak peak is likely related to stacking faults or polytype intergrowth (e.g., 4H-SiC) [51,52]. Despite these deviations, the main diffraction peaks remain in good agreement with the standard reference pattern of 6H-SiC (PDF#74-1302), indicating that the primary crystalline phase of the sample is still 6H-SiC.

**Figure 2:**
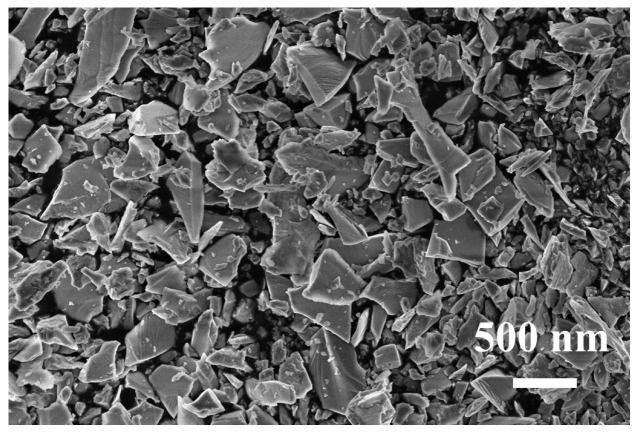
SEM image of the SiC particles.

The surface morphology structure of the SiC particles is examined using SEM. As shown in Figure 2, the SiC particles have an irregular block structure with non-uniform sizes and small fine particles attached to their surface, which can increase the surface roughness and mechanical strength of the contact surfaces during tribocatalysis, thereby promoting the generation of more charges for the decomposition of dyes.

**Figure 3:**
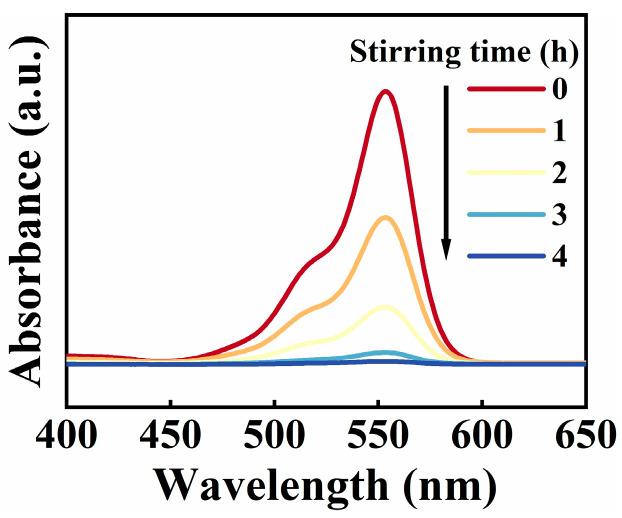
The RhB dye absorption spectra using these SiC particles at 600 rpm for 4 h.

The RhB decomposition process, illustrated in Figure 3, is performed with a PTFE rotating disk set at 600 rpm. The absorption intensity at 554 nm, characteristic of RhB, diminishes with time, demonstrating the decomposition process. The decomposition ratio (*D*) is determined according to Equation (1) [53]:

(1) $$D=(1-A_{t}/A_{0})\times 100\%$$

Here, *A*_0_ denotes the initial absorbance intensity of the RhB solution prior to tribocatalytic processing, while *A*_t_ denotes the absorbance recorded after a given reaction time, *t.*

**Figure 4:**
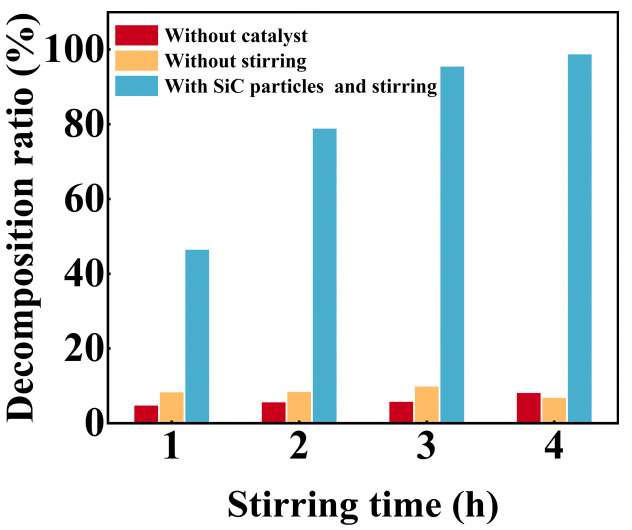
The decomposition of RhB dye using the SiC particles under various reaction conditions.

According to Figure 4, the decomposition ratio of RhB dye is negligible, without the addition of SiC particles. As illustrated in Figure 4, after stirring for 4 h, the decomposition ratios of RhB dye are 98.9%, 8.3% and 7.0% with both the catalyst and stirring, with stirring but without the catalyst and with the catalyst but without stirring, respectively. The decomposition ratio of RhB dye in the presence of the catalyst but without stirring is almost negligible, indicating that the catalyst exhibits a poor decomposition ratio of RhB dye. A comparison of the RhB decomposition ratios under stirring conditions, with and without the catalyst, clearly demonstrates that the catalyst markedly improves the dye decomposition efficiency. This finding indicates that the SiC particles are essential for the tribocatalytic decomposition of dyes.

**Figure 5:**
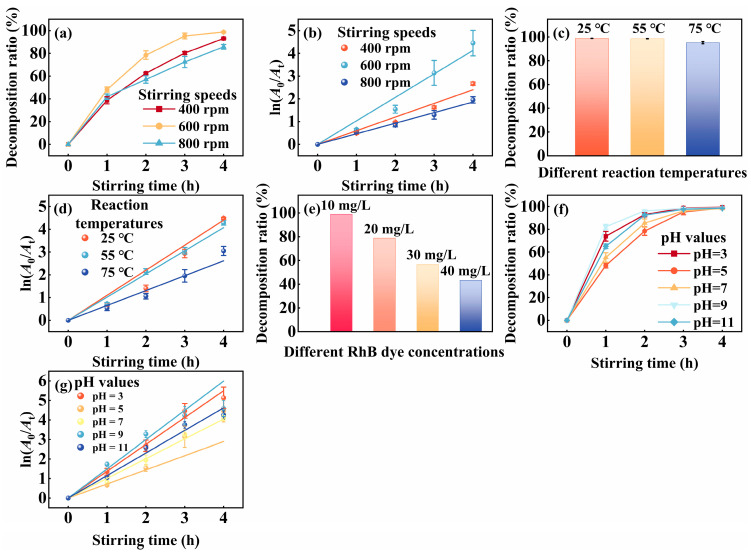
The RhB decomposition ratios are presented for (**a**) different stirring speeds, (**c**) reaction temperatures, (**e**) dye concentrations, and (**f**) pH values. The corresponding pseudo-first-order rate constants, *K*, (h^−1^) are shown in (**b**) stirring speeds, (**d**) reaction temperatures, and (**g**) pH values.

The effect of stirring intensity on RhB dye decomposition is examined in the presence of 200 mg of the SiC particles. As illustrated in Figure 5a, the decomposition ratio initially increased with increasing stirring strength, reached a maximum at an intermediate level, and then declined under the most vigorous condition. This non-monotonic behavior is further confirmed by the first-order kinetic fitting in Figure 5b, where the ratio constant *K* exhibits a similar trend. Specifically, the *K* value increases by approximately 72% from the lowest to the optimal stirring condition and subsequently decreases by ~54% at the highest level. These variations exceed the respective standard deviations (Table 1), suggesting that the observed differences are statistically significant.

The close correspondence between the trend in decomposition efficiency and the kinetic constants implies that tribocatalytic activity is highly sensitive to mechanical input. Moderate stirring likely enhances interfacial contact between SiC and the PTFE surface, promoting effective charge separation and radical generation. In contrast, under vigorous stirring, excessive mechanical friction may accelerate charge dissipation before sufficient reactive species accumulate, thereby reducing overall catalytic efficiency [54]. Moreover, strong vortex flows may cause the SiC particles to migrate toward the container walls, diminishing their interaction with the active surface and forming ‘dead zones’ that limit effective collisions [26,55]. This interpretation is supported by the curvature in Figure 5a and the reduced slope of the linear fit in Figure 5b under extreme stirring, both of which reflect diminished reaction rates at high shear levels. The corresponding kinetic parameters and decomposition results are summarized in Table 1.

A systematic investigation is carried out to evaluate the influence of temperature on dye decomposition under tribocatalytic conditions. As shown in Figure 5c, the decomposition efficiency remains relatively stable across the tested temperature range, with only a modest decline observed at elevated temperatures. This trend is further supported by the first-order kinetic fitting results in Figure 5d, where rate constant *K* exhibits a slight increase from 25 °C to 55 °C (1.00→1.04 h^−1^), followed by a marked reduction at 75 °C (0.69 h^−1^). The overall decrease in *K* from 55 °C to 75 °C corresponds to a ~34% decline, which significantly exceeds the associated standard deviations (Table 1), suggesting a statistically meaningful effect of temperature on catalytic activity.

These findings imply that tribocatalytic dye decomposition over SiC is not strongly thermally activated and primarily depends on mechanical charge generation. However, the diminished performance at elevated temperatures may be linked to thermally induced changes in the PTFE surface. Specifically, increased temperature can reduce the dielectric constant and promote surface defect formation, both of which may impair triboelectric charge separation [56]. Additionally, the thermal softening of the PTFE matrix likely reduces surface hardness and elastic modulus, limiting frictional abrasion from SiC particles and thus decreasing charge carrier generation efficiency [57]. The fitted kinetic parameters for different temperature conditions are summarized in Table 1.

The effect of different RhB dye concentrations on tribocatalytic dye decomposition is investigated, as illustrated in Figure 5e. A gradual decrease in the RhB decomposition ratio is observed with increasing initial dye concentration: 98.9% (10 mg/L), 78.9% (20 mg/L), 56.6% (30 mg/L), and 43.3% (40 mg/L). A gradual decrease in decomposition efficiency is observed with increasing dye concentration. This trend may be attributed to the limited generation of reactive species within the fixed stirring duration, which might become insufficient to fully decompose higher dye loads. At lower concentrations, the number of active radicals may be adequate for efficient decomposition, whereas at higher concentrations, excessive dye molecules could compete for or consume reactive species more rapidly. In addition, increased solution opacity and surface adsorption at high concentrations might hinder the interaction between the SiC particles and dye molecules, potentially limiting charge transfer and radical diffusion. These factors together may contribute to the reduced catalytic performance observed at higher dye concentrations.

The influence of pH on the tribocatalytic decomposition of RhB is investigated across a wide pH range. As shown in Figure 5f, the overall decomposition efficiency remained consistently high under both acidic and alkaline conditions, with only minor variations. However, the kinetic analysis in Figure 5g reveals subtle differences in the reaction rate constants, *K*, which suggest that the interfacial behavior of the catalyst may be influenced by solution pH. Specifically, *K* reaches its highest value at pH = 3 (1.10 h^−1^), decreases to a minimum at pH = 7 (0.88 h^−1^), and increases again under alkaline conditions (1.02 h^−1^ at pH = 11). The difference between the maximum and minimum values represents a ~20% change in ratio constant, exceeding the corresponding standard deviations (Table 1), thus indicating a statistically meaningful modulation of catalytic activity by pH.

These results imply that, although the overall decomposition efficiency is not highly pH-sensitive, the initial reaction kinetics are responsive to pH-induced surface modifications. In acidic media, the protonation of surface groups and formation of hydroxyl or carboxyl functionalities may enhance dye adsorption and charge accumulation. In contrast, under alkaline conditions, the partial etching of the native SiO_x_ layer and formation of polar groups could facilitate interfacial electron transfer [58]. Near-neutral conditions may result in a more inert surface state, reducing interfacial charge activity and slightly lowering the reaction rate [59]. These observations suggest that acid–base-induced surface regulation plays a secondary, yet noticeable, role in tribocatalytic performance. The fitted kinetic data are presented in Table 1.

**Figure 6:**
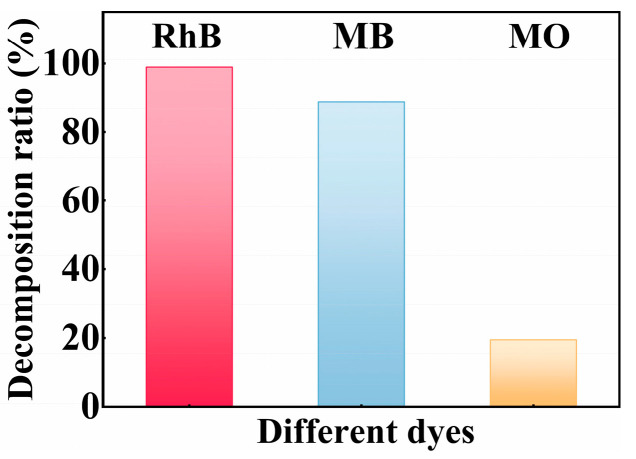
The tribocatalytic decomposition ratio of different dyes using the SiC particles.

The versatility of the SiC particles is explored through the decomposition of various dyes. As illustrated in Figure 6, the decomposition ratios for various dyes are approximately 98.9% for Rhodamine B (RhB), 88.7% for Methyl Blue (MB), and 19.5% for Methyl Orange (MO), respectively. As a matter of fact, the decomposition of dyes is a process that breaks the chemical bonds of dye molecules. However, the energy required to cleave various chemical bonds differs depending on bond type. Weak chemical bonds, such as C–N and C–C, are more effectively broken, whereas bonds with high-energy, like N=N and C=N, require a significant amount of energy to break [60,61,62]. Among these three dyes, RhB is easily decomposed because of its relatively low-energy bond [63].

The decomposition ratio of MO in the tribocatalytic system is relatively low, which is the result of a combination of various mechanisms, including the strong stability of its molecular structure, significant charge repulsion, weak interfacial adsorption ability, and competitive reaction disadvantages [64]. To enhance the decomposition performance of such azo dyes, strategies such as introducing co-catalysts, optimizing the interfacial electric field structure, or adjusting pH conditions may be considered [65].

**Figure 7:**
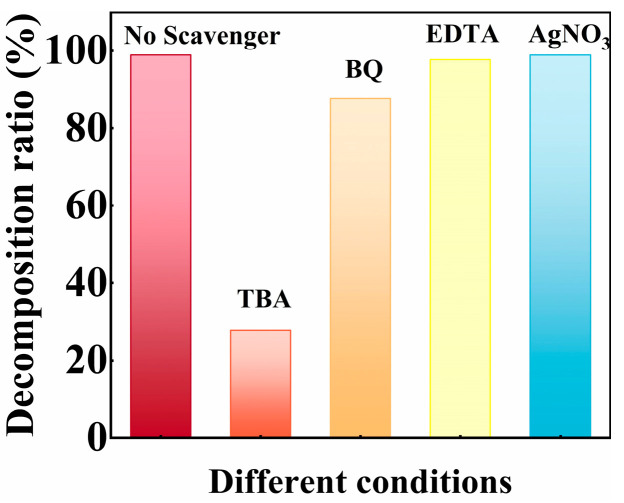
The decomposition ratio of RhB dye using the SiC particles in the presence of TBA, BQ, EDTA, and AgNO_3_ scavengers.

To investigate the dominant reactive species generated by SiC particles during the tribocatalytic reaction, EDTA, TBA, BQ, and AgNO_3_ are individually added as scavengers into the RhB dye solution. According to Figure 7, the dosing of BQ and TBA—used to scavenge superoxide radicals (·O_2_^−^) and hydroxyl radicals (·OH), respectively—leads to a pronounced decline in decomposition efficiency, indicating that these two species play key roles in the tribocatalytic process. In contrast, when EDTA and AgNO_3_ are employed to scavenge photogenerated holes (h^+^) and electrons (e^−^), respectively, the RhB decomposition ratio exhibits negligible variation compared to the control group, suggesting that h^+^ and e^−^ are not the principal contributors in this system.

**Figure 8:**
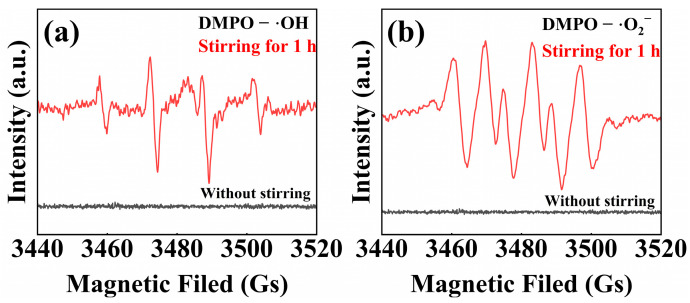
The EPR spectrum of (**a**) DMPO spin-trapping for ·OH; and (**b**) DMPO spin-trapping for ·O_2_^−^.

To further verify the evolution of ·OH and ·O_2_^−^ radicals in the course of the tribocatalytic process, EPR spectroscopy is employed to detect these key reactive species. As presented in Figure 8a,b, after 1 h of mechanical agitation, four characteristic peaks corresponding to DMPO–·OH and six peaks attributed to DMPO–·O_2_^−^ adducts are observed, thereby confirming the generation of hydroxyl and superoxide radicals [66].

In the process of tribocatalysis, the SiC particles with high hardness rubbed against the PTFE rotating disk, generating scratches and nanoscale defects on the PTFE surface. This mechanical interaction not only physically increases the roughness and effective contact area of the catalytic interface but also contributes to enhanced electron excitation and charge transfer at the interface. The grinding and tribocatalytic mechanisms synergize during the reaction, promoting the efficient decomposition of organic dye molecules.

On the one hand, the mechanical friction continuously forms micro- and nano-scratches on the PTFE rotating disk surface through applied shear forces. These scratches enlarge the active surface area and expose reactive edge sites of PTFE, which can interact more effectively with reactant species. Additionally, the mechanical abrasion process may produce loose PTFE fragments or particles, which further participate in charge redistribution and radical generation in the reaction zone.

On the other hand, the scratches generated on the hard SiC particle surfaces may lead to localized charge accumulation due to surface polarization and defect formation. Given the excellent electronegativity of PTFE, during sliding contact, negative charges tend to accumulate on the PTFE rotating disk while positive charges concentrate on the SiC surface, leading to an asymmetric electric field across the interface. This built-in field enables anisotropic electron transport across the PTFE–SiC interface, modulated by sliding direction and surface characteristics.

The spatial separation of charges across the PTFE/SiC interface plays a critical role in initiating redox reactions. Triboelectrically induced charge carriers are capable of initiating redox reactions with ambient water and oxygen, giving rise to the emergence of ROS such as ·OH and ·O_2_^−^. These ROS are highly oxidative and capable of attacking and breaking down complex organic dye molecules into smaller, less harmful fragments.

In addition, the positive charges generated on the SiC surface due to friction activation may enhance its adsorption of negatively charged dye molecules or their intermediate free radicals. As a result, reactants become concentrated near the catalytic sites, which intensifies interfacial reactions and facilitates the cleavage of chemical bonds in dye compounds by lowering the activation threshold.

**Figure 9:**
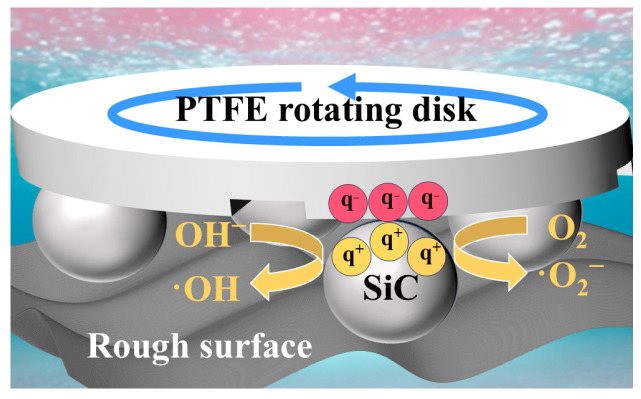
Tribocatalytic mechanism of the SiC particles.

Figure 9 illustrates a schematic representation of the tribocatalytic dye decomposition mechanism mediated by the SiC particles. The underlying reaction mechanisms governing tribocatalytic dye decomposition are represented by Equations (2)–(5):

(2) $$SiC\;+PTFE\;\overset{Friction}{\to }\;SiC(q^{+})+PTFE(q^{-})$$

*q^−^* + *O_2_* → *·O_2_^−^* (3)

*q^+^* + *OH^−^* → ·*OH* (4)

*·O_2_^−^* or ·*OH + RhB* → *Decomposition* (5)

**Figure 10:**
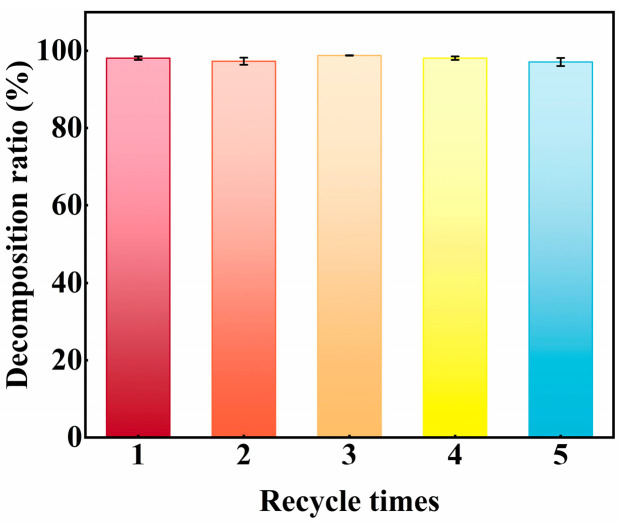
The RhB dye decomposition ratio of the SiC particles in the cycling experiment.

To evaluate its practical viability, the catalyst’s recycling stability is thoroughly examined through several reaction cycles. As depicted in Figure 10, the catalyst preserves stable tribocatalytic dye decomposition performance, with no significant reduction in activity after five continuous cycles, underscoring its remarkable recyclability and suitability for real-world applications.

## 4. Conclusions

In summary, the decomposition of RhB dye is driven by the utilization of physical agitation energy generated through friction. The experimental results clearly demonstrate that the observed tribocatalytic activity originates from the interfacial contact between the SiC particles and the PTFE rotating disk. The SiC particles were stirred for 4 h (600 rpm) to decompose RhB dye and the decomposition ratio was up to ∼98.9%. Both the quenching experiment of electron spin resonance analysis and active species detection jointly indicate that hydroxyl radicals (·OH) and superoxide anions (·O_2_^−^) function as the principal reactive species entities in the system. Due to their excellent tribocatalytic performance, the SiC particles can be used to harvest friction energy for water purification.

The fixed porous silicon carbide structure is emerging as a highly promising friction catalytic material due to its high surface area, mechanical strength, thermal stability, and chemical resistance [67,68]. Unlike particle catalysts, porous silicon carbide exhibits excellent recyclability, structural stability, and enhanced water interface contact, thereby improving catalytic efficiency. Its porous structure also facilitates integration into scalable continuous flow processing systems. In summary, these characteristics make porous silicon carbide an ideal choice for long-term, stable, and efficient friction catalytic applications in the field of environmental remediation.

## Figures and Tables

**Table 1:** Average decomposition ratios and pseudo-first-order rate constants *K* (h^−1^) of RhB under different experimental conditions. Each value represents the mean ± standard deviation from three independent experiments.

Condition Type	Parameter	Decomposition (%)	*K* (h^−1^)
**Stirring Speeds (rpm)**	400	93.05 ± 0.50	0.60 ± 0.04
	600	98.70 ± 0.73	1.03 ± 0.07
	800	85.74 ± 2.05	0.47 ± 0.02
**Reaction Temperatures (°C)**	25	98.87 ± 0.05	1.10 ± 0.04
	55	98.61 ± 0.13	1.02 ± 0.06
	75	95.20 ± 0.90	0.65 ± 0.05
**pH Values**	3	99.35 ± 0.30	1.37 ± 0.04
	5	98.70 ± 0.73	0.72 ± 0.07
	7	98.33 ± 0.24	1.01 ± 0.04
	9	98.92 ± 0.46	1.50 ± 0.12
	11	98.56 ± 0.29	1.15 ± 0.05

## Data Availability

The data presented in this study are available in this article.
